# The influence of crystal structures on the performance of CoMoO_4_ battery-type supercapacitor electrodes†

**DOI:** 10.1039/d3ra05878f

**Published:** 2024-03-11

**Authors:** Kunli Yang, Joseph P. Cline, Bohyeon Kim, Christopher J. Kiely, Steven McIntosh

**Affiliations:** a Department of Chemical and Biomolecular Engineering, Lehigh University Bethlehem PA 18015 USA mcintosh@lehigh.edu; b Department of Materials Science and Engineering, Lehigh University Bethlehem PA 18105 USA

## Abstract

CoMoO_4_ is a promising battery-type supercapacitor electrode material that can offer relatively high storage capacity and cycle stability. In this work, we investigate the role of the crystalline phase of CoMoO_4_ in determining these performance parameters. The hydrate phase of CoMoO_4_ was synthesized on a nickel foam substrate *via* hydrothermal reaction with subsequent annealing under an inert atmosphere leading to the formation of the β-phase CoMoO_4_. Similar nanoplate morphologies were observed in all of the samples. The hydrate-phase CoMoO_4_ demonstrates larger specific capacity than the annealed β-phase CoMoO_4_. Besides, the samples synthesized at lower temperatures have better rate capability than the sample annealed at higher temperatures. However, the hydrate phase had worse long-term stability compared to the β-phase samples.

## Abstract 2

The low-temperature hydrate phase of CoMoO_4_ exhibits facile surface kinetics and high capacity as a supercapacitor electrode material.

## Graphical abstract

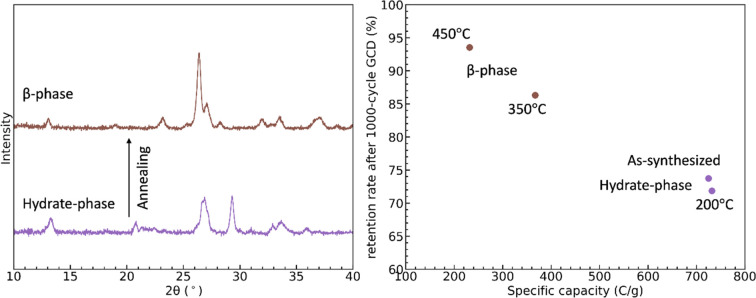

## 1. Introduction

The intermittent nature of renewable energy requires the further development of energy storage technologies, with coupling between these energy storage methods likely necessary to meet demands for both high power and high energy storage capacity.^1,2^ Supercapacitors are of great interest as they can potentially provide substantially higher power density than battery systems, increased capacity in comparison with traditional film capacitors, and long cycle life.^3^ Based on their charge storage mechanisms, supercapacitors can be broadly classified as electrical double-layer capacitors (EDLCs) or pseudocapacitors. EDLCs store charge *via* a non-faradaic process whereby charges are electrostatically separated and are adsorbed onto the surfaces of the electrodes. Therefore, the EDLC capacity is proportional to the electrode surface area,^4^ providing very high-power density but only limited energy density.^3^ Compared to EDLCs, pseudocapacitors store energy through fast near-surface redox reactions to provide higher specific capacitance^5,6^ while maintaining high power density.^7,8^

RuO_2_ was first introduced as the electrode material for pseudocapacitors because of its multiple oxidation states, wide electrical potential range, high conductivity, and superior electrochemical reversibility.^9–12^ However, the scarcity of resources and the extremely high cost of RuO_2_ hinders wide application.^13,14^ The report of the pseudocapacitive behavior of RuO_2_ inspired the exploration of other TMOs, such as cobalt oxide, nickel oxide, and iron oxide, with manganese oxide receiving the most interest.^3,15–22^ Even with many advantages such as multiple oxidation states, high abundance, low cost, and environmental benignity, several shortcomings of TMOs like poor electrical conductivity still need to be addressed.^23^ More recently, transition metal molybdates (TMMs), especially NiMoO_4_ and CoMoO_4_ are emerging as excellent electrode materials, due to their electrochemical activity, enhanced electronic conductivity, and high abundance.^24–26^ To enhance the electrochemical properties of CoMoO_4_, approaches including fabricating unique morphologies and making composites with conductive carbon materials have been demonstrated.^27–31^

In this article, we studied the influence of annealing temperature on the electrochemical properties of CoMoO_4_. CoMoO_4_ was synthesized on nickel foams *via* a facile hydrothermal reaction. Hydrate-phase CoMoO_4_ was obtained at lower annealing temperatures, while β-phase CoMoO_4_ was synthesized at 350 °C and higher temperatures. The hydrate-phase CoMoO_4_ has higher specific capacity than the β-phase CoMoO_4_. The long-term stability of CoMoO_4_ is also closely related to the crystal phase. The decrease in electrochemical surface area (ECSA), increased charge transfer resistance, and worse diffusion are the main factors that lead to the degradation during long-term tests. Besides, 350 °C CoMoO_4_ with lower crystallinity has a higher specific capacity than 450 °C CoMoO_4_ which has a higher crystallinity, indicating that crystallinity also influences the electrochemical properties of CoMoO_4_.

## 2. Experimental

### 2.1 Synthesis of CoMoO_4_ on nickel foam substrate

CoMoO_4_ was synthesized on nickel foams^32–34^ by a facile hydrothermal method. Before the hydrothermal reaction, a piece of nickel foam (3 cm × 2 cm × 0.1 cm) was cleaned by ultrasonication in ethanol and deionized water. 1.5 mmol cobalt nitrate hexahydrate (Co(NO_3_)_2_·6H_2_O) and 1.5 mmol sodium molybdate dihydrate (Na_2_MoO_4_·2H_2_O) were dissolved in 30 mL DI water and stirred for 30 minutes to form a clear purple solution. A cleaned nickel foam was immersed in the solution within an autoclave prior to sealing and heating in an oven at 160 °C for 4 hours. The sample was then cleaned by ultrasonication in deionized water and dried overnight in an oven at 60 °C. Without further treatment, such samples are labeled herein as-synthesized CoMoO_4_. The weight of nickel foam before and after the hydrothermal reaction was carefully measured, and the increased weight was regarded as the weight of active materials. The average loading of CoMoO_4_ on the nickel foam for all samples was between 0.90 and 0.92 mg cm^−2^. To study the influence of the annealing temperatures, some as-synthesized samples were annealed under N_2_ environment at 200 °C, 350 °C, and 450 °C for 2 hours, and are herein labeled as such.

### 2.2 Characterization

The crystalline structures of the samples were studied by X-ray diffraction (XRD, Cu Kα radiation, PANalytical Empyrean X-ray Diffractometer). The interlayer spacing of the samples was calculated by Bragg's law, eqn S1,† using the data extracted from XRD. Thermogravimetric analysis (TGA, TA instrument Q500) was applied to study the water loss of CoMoO_4_ during annealing. TGA curves were collected from 25 °C to 500 °C under N_2_ atmosphere at a heating rate of 5 °C min^−1^. Raman spectra were collected (WITec Raman Imaging alpha300 R) with a 532 nm wavelength laser. The morphologies of the samples were imaged by field emission scanning electron microscopy (FESEM, Hitachi 4300 SE) with accompanying elemental analysis by X-ray energy dispersive spectrometry (XEDS). Transmission electron microscopy (TEM) was applied to study the crystal structure of observed morphologies.

Electrochemical measurements, including cyclic voltammetry (CV), galvanostatic charge–discharge (GCD), electrochemical impedance spectroscopy (EIS), and electrochemical surface area (ECSA) were carried out in a traditional three-electrode system (Gamry Reference 3000, Gamry Instruments, Malvern PA, USA), using 2 M KOH aqueous solution as the electrolyte, Pt mesh as the counter electrode, and Hg/HgO reference electrode. CV curves were measured between 0 and 0.6 V at scan rates between 1 mV s^−1^ and 10 mV s^−1^. The CV curves typically stabilized after 20 cycles. Information on calculation of calculating the electrode capacity, ESCA, and fitting of the cyclic voltammograms can be found in the ESI.†

## 3. Results and discussion

### 3.1 Structural characterization

**Fig. 1:**
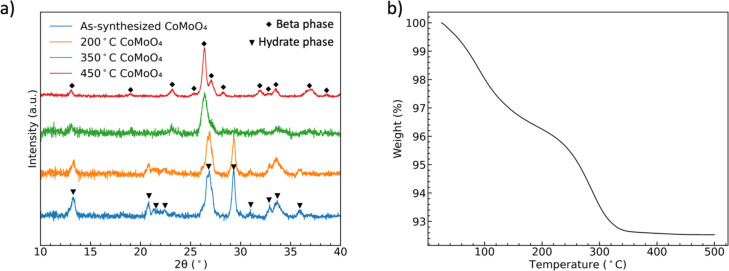
(a) XRD patterns showing the formation of two phases of CoMoO_4_, (b) TGA curves of the hydrate CoMoO_4_ from 25 °C to 500 °C in N_2_ showing the two processes of dehydration.

Fig. 1(a) shows the XRD patterns of CoMoO_4_ on Ni mesh as-synthesized and after annealing in N_2_ at 200 °C, 350 °C, and 450 °C for two hours. The XRD patterns of as-synthesized and 200 °C annealed CoMoO_4_ match well with standard patterns of the triclinic hydrate phase of CoMoO_4_ (PDF #14-0086), while the XRD patterns of samples annealed at 350 °C and 450 °C CoMoO_4_ match with the standard patterns of monoclinic β-phase CoMoO_4_ (PDF #21-0868). The sharper peaks of 450 °C annealed CoMoO_4_ indicate that, as expected, the crystallites grow as the annealing temperature increases. The color of the materials changed from purple to brown as the hydrate phase transformed into the β-phase.

Both CoMoO_4_ phases have octahedrally coordinated CoO_6_ and tetrahedrally coordinated MoO_4_, with the hydrate phase having additional coordination water bound to Co atoms.^35^ The CoO_6_ octahedra form two different [CoO_6_]_4_ units depending on the phases: in the β-phase, edge-sharing CoO_6_ octahedra can be considered closely packed, while in the hydrate-phase the CoMoO_4_, are more loose-packed in a Z-shape [CoO_6_]_4_ unit. The lattice water in the hydrate phase occupies the interstitial sites in this more open structure.^35^ The interlayer spacing of the hydrate phase and β phase is calculated by Bragg's law based on the XRD data, Fig. S1.† The calculated interlayer spacing matches with the two crystalline structures, also confirming the more open structure of hydrate phase.^35–37^ The overall similarity of metal coordination enables the facile transition from hydrate- to β-phase upon annealing.^35^

TGA of as-synthesized material under N_2,_Fig. 1(b), indicates two distinct weight loss regions in the temperature range of 60 °C to 200 °C and 240 °C to 340 °C.^38^ The first weight loss is generally attributed to the reversible loss of lattice water, while the second dehydration is attributed to the irreversible phase change from hydrate- to β-phase, as indicated by the XRD patterns. We noted in our own experiments that samples heated to only 200 °C would regain mass is left in the open laboratory for a period of several hours, indicating that the samples can reabsorb water from the environment. However, the weight loss was permanent after annealing at and above 350 °C.

**Fig. 2:**
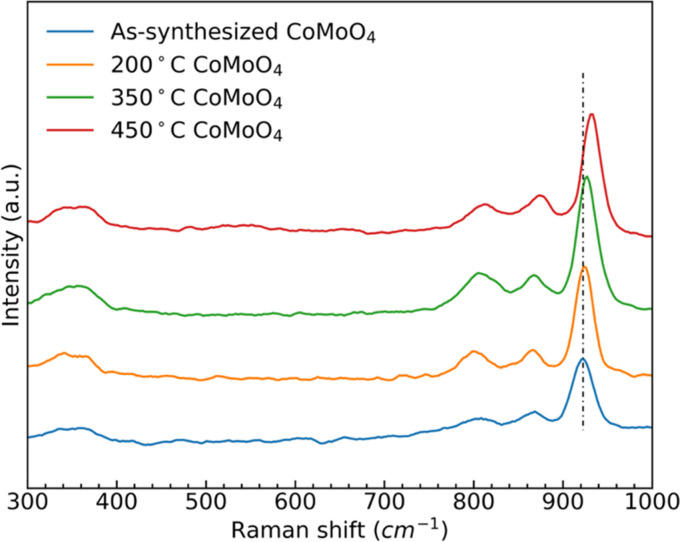
Raman spectra of as-synthesized, 200 °C, 350 °C, and 450 °C CoMoO_4_.

Raman spectra of the as-synthesized and annealed samples, Fig. 2, show three similar bands between 950 cm^−1^ and 800 cm^−1^, and two bands around 350 cm^−1^. The strong band at 930 cm^−1^ and weaker bands at 880 cm^−1^ and 350 cm^−1^ all correspond to the stretching vibrations of Mo–O–Co in CoMoO_4_.^39,40^ The peak at 340 cm^−1^ is due to the vibration of MoO_4_.^41^ After the transition from the hydrate phase to the β phase, the stronger peak at around 930 cm^−1^ shifted to a higher wavenumber, due to the reduced Mo–O–Co bond length in the β-phase CoMoO_4_.

**Fig. 3:**
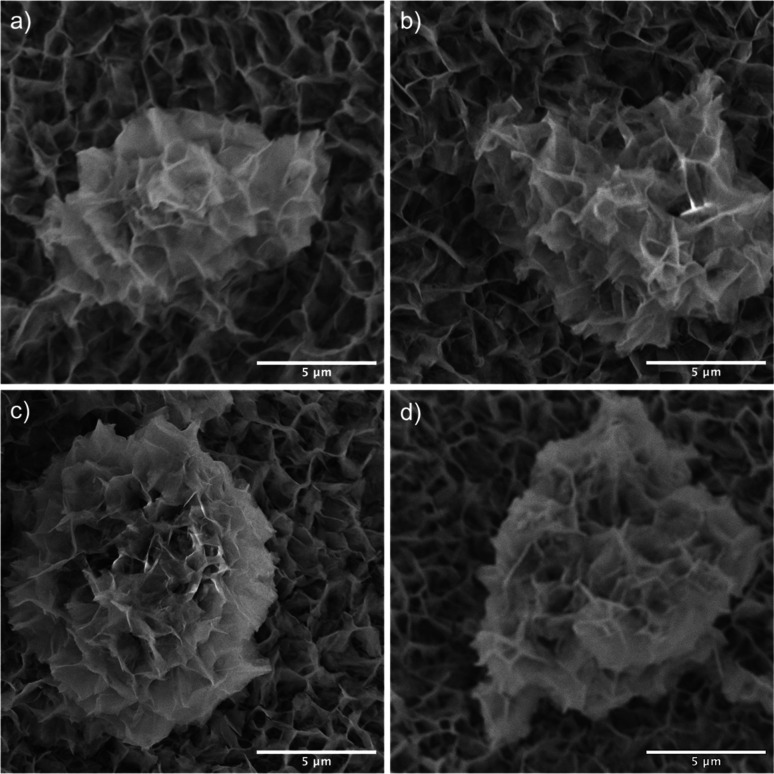
SEM images of (a) the as-synthesized, (b) 200 °C, (c) 350 °C, and (d) 450 °C samples showing that the nanoplates cover the surface of nickel foam with some large nanoplate agglomerates existing within each sample.

**Fig. 4:**
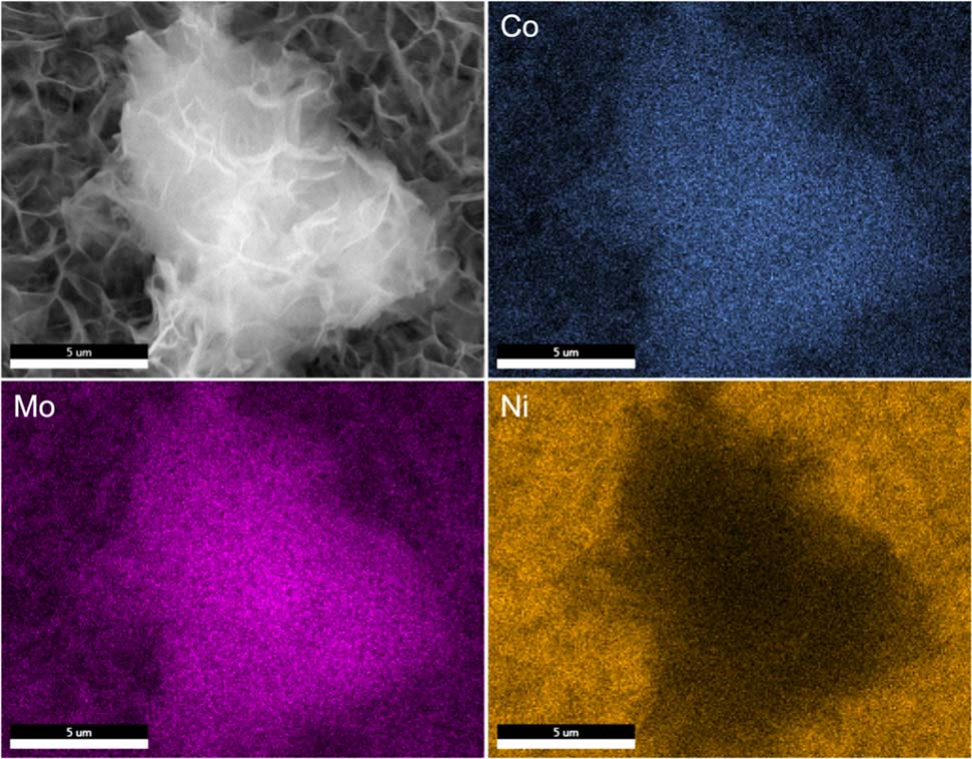
EDS mapping of as-synthesized CoMoO_4_ showing the presence of Co, Mo, and Ni elements.

SEM images reveal that the synthesis yields a thin film of CoMoO_4_ nanoplates over the surface of the nickel foam with associated agglomerations of CoMoO_4_ nanoplates growing on the surface of the thin film, Fig. 3. Lower magnification wider views are provided in Fig. S2.† This morphology is maintained after annealing despite the change in the crystal structure, likely due to the overall structural similarity^35^ and small unit cell volume change from 103.8 Å^3^ of hydrate-phase CoMoO_4_ to 106.5 Å^3^ β-phase CoMoO_4_. Associated EDS mapping confirms the uniform distribution of Co and Mo throughout these structures as synthesized, Fig. 4. The high background Ni signal is due to the Ni-foam substrate and is lower in the region of the larger central nanoplate agglomeration due to lower Ni concentration relative to the Ni-foam. A sample of the as-synthesized material was mechanically removed from the nickel film for TEM analysis. Fig. S3(a)† is a TEM image of the removed nanoplate with the associated electron diffraction pattern shown in Fig. S3(b),† with the crystal structure of these morphologies matching the hydrate phase, Table S1.†

### 3.2 Electrochemical characterization

**Fig. 5:**
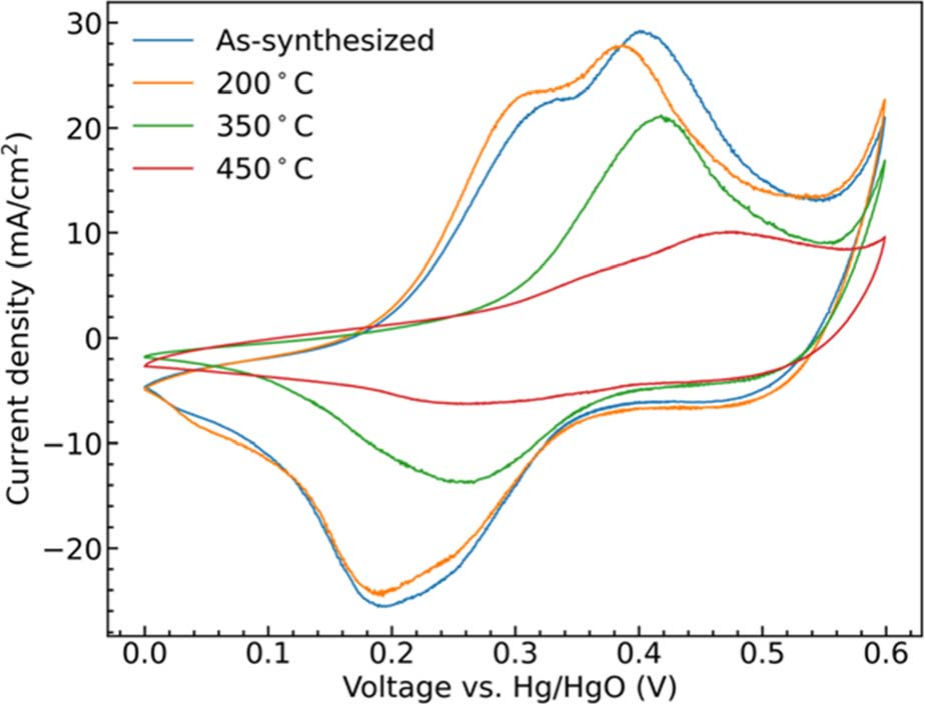
CV curves of as-synthesized CoMoO_4_, 200 °C CoMoO_4_, 350 °C CoMoO_4,_ and 450 °C CoMoO_4_ from 0 to 0.6 V (*vs.* Hg/HgO) at a scan rate of 10 mV s^−1^ in 2 M KOH.

Cyclic voltammetry (CV) collected from the samples show clear redox peaks in the measured potential window, Fig. 5, demonstrating that the CoMoO_4_ structures are active as possible pseudocapacitor materials in alkaline electrolyte. These redox peaks are attributable to changes in Co oxidation state as Mo is not considered to be redox active within this window. Mo is suggested to aid in both enhancing electronic conductivity and providing structural integrity to the electrode material when compared to the use of pure cobalt oxide.^42,43^ The two hydrate phase electrode materials, the as-synthesized CoMoO_4_ and 200 °C CoMoO_4_ show two distinct oxidation peaks centered around 0.31 V and 0.39 V (*vs.* Hg/HgO). This indicates that the hydrate-phase CoMoO_4_ has two distinct and accessible oxidation states or structures of Co. Given that the Co within the hydrate phase of CoMoO_4_ exists in a similar octahedral coordination with similar Co–O bond distances,^35^ we may anticipate that these distinct oxidation peaks are due to distinct changes in oxidation state rather than crystallographic site dependent. Based on prior computational reports, we suggest that the first of these peaks is attributable to Co^2+^ being oxidized to Co^3+^ at a lower potential, prior to further oxidation to Co^4+^ as the potential increases.^44^

The two β-phase materials, those annealed at 350 °C and 450 °C, show less single oxidation and reduction peaks and are generally shifted to higher potentials at a scan rate of 10 mV s^−1^. The 350 °C annealed sample shows a single peak, likely due to the overlapping of the redox potentials of the two redox transitions. The 450 °C shows two overlapping, lower intensity, but still distinct peaks shifted to higher potential by around 0.07 V when compared with the as-synthesized hydrate-phase sample. At the same scan rate, the area within the CV curves is proportional to the capacitance of the electrode indicating that the hydrate-phase CoMoO_4_ has a larger capacitance than the β-phase CoMoO_4_.

The shifting of the redox potentials as the crystal phase transitions from the hydrate phase to the β-phase is attributable to the change of the Madelung electric field exerted on the metal ions. With the redox potential increasing when the field becomes weaker.^45,46^ For example, Padhi *et al.* explained that the redox potential of Fe^2+/3+^ in an olivine structure was higher than that in a NASICON structure due to a reduction in the Madelung electric field experienced by Fe cations in the edge-sharing FeO_6_ octahedra within the olivine structure.^47^ Similarly, in CoMoO_4_, Co cations in the more compact edge-sharing β-phase of CoMoO_4_ will experience a reduced field, and thus demonstrate a higher redox potential when compared to the hydrate phase.^35^

**Fig. 6:**
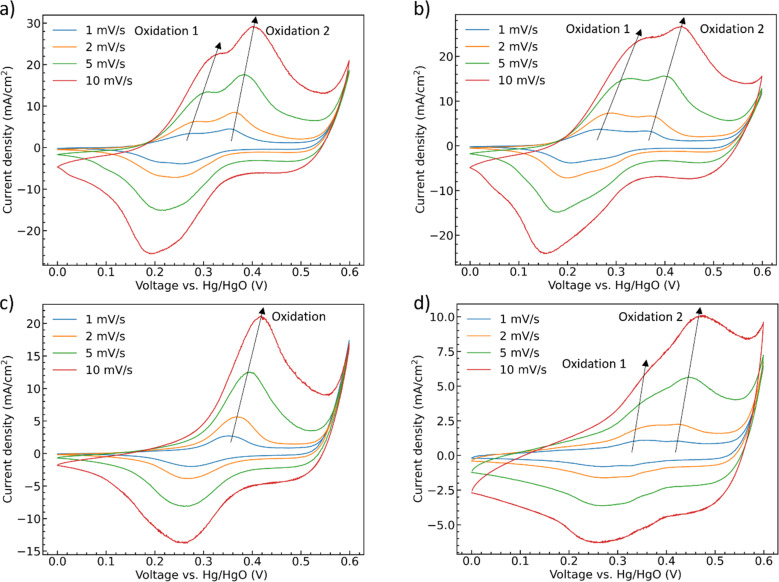
CV curves as a function of scan rate (a) as-synthesized CoMoO_4_, (b) 200 °C CoMoO_4_, (c) 350 °C CoMoO_4_, (d) 450 °C CoMoO_4_ from 0 to 0.6 V (*vs.* Hg/HgO) in 2 M KOH.

To further deconvolute the redox behavior of the materials, CV scans were performed at scan rates from 1 mV s^−1^ to 10 mV s^−1^, Fig. 6. The oxidation and reduction peaks of CoMoO_4_ shift to higher and lower potential, respectively, as the scan rate increases as expected from standard kinetic models.^32^ The total charge storage mechanism of pseudocapacitors can generally be considered as a combination of surface and bulk processes.^48^ These are typically deconvoluted through the simple approach proposed by Linström *et al.*^49^ where the peak current density of the redox reaction is related to the scan rates by a power-law relationship with pre-exponential, a, and exponent, *b*, eqn S2.†^50^ The exponent “*b*” can vary between 0.5 for a bulk-limited process to 1.0 for a surface-limited process. The *b* values of the four CoMoO_4_ samples are between 0.5 and 1, Table S3,† indicating that the charge storage mechanism is a mixture of the two processes, noting that the *b* values for the hydrate-phase are smaller than for β-phase. This result suggested that more of the bulk material is involved in the redox reactions for the hydrate phase, at least partly explaining the demonstrated larger specific capacity compared to the more surface limited β phase.

The current contribution from surface and bulk can be further assessed by the following equation:^51–55^*i*(*v*) = *k*_1_*v* + *k*_2_*v*^1/2^ = *i*_surface_ + *i*_bulk_

**Fig. 7:**
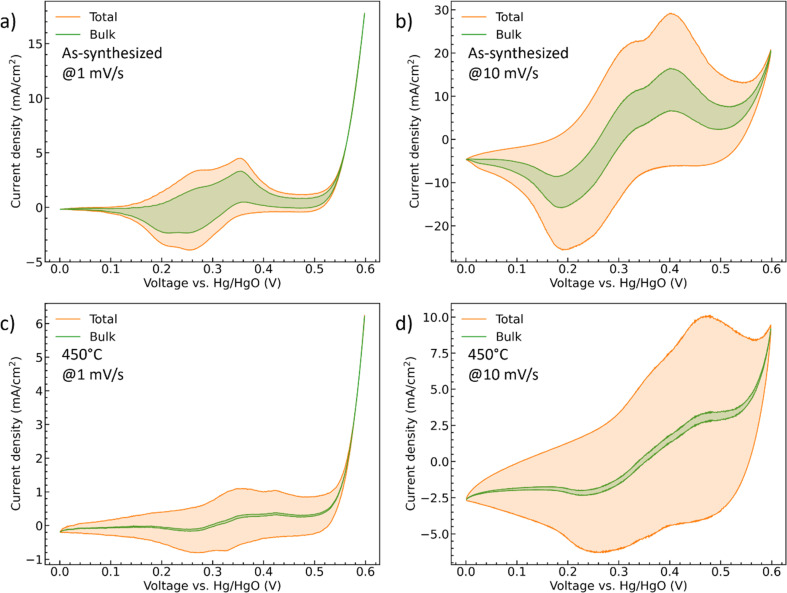
The CV curves of (a and b) the as-synthesized and (c and d) 450 °C CoMoO_4_ with deconvoluted diffusion-controlled current contributions at both 1 mV s^−1^ and 10 mV s^−1^.

*i*(*v*) is the peak current density (A cm^−2^) at different scan rate *v* (mV s^−1^), *k*_1_ (A cm^−2^ mV^−1^ s) and *k*_2_ (A cm^−2^ mV^−1/2^ s^1/2^) are the constants, *i*_surface_ (A cm^−2^) and *i*_bulk_ (A cm^−2^) stand for the contribution of the capacitive- and diffusion-controlled process. Therefore, by plotting and fitting *i*/*v*^1/2^*vs. v*^1/2^, the value of constant *k*_1_ (the slope) and *k*_2_ (the *y* intercept) can be determined, Fig. S4.† For the as-synthesized hydrate-phase sample, Fig. 7(a) and (b), the current contribution of the bulk decreased from 54.76% to 27.68% when the scan rate increased from 1 mV s^−1^ to 10 mV s^−1^. This was because more diffusion-controlled bulk reaction was involved when the scan rate was slow. Similarly, in the 450 °C sample, Fig. 7(c) and (d), the current contribution of the bulk decreased from 12.36% to 4.27%. The bulk portion in the 450 °C sample was significantly smaller than that in the as-synthesized sample, which matched the conclusion made in the discussion of the *b* values. We suggest that this greater bulk activity is due to the more open crystal structure and potential for ionic transport within the hydrate phase.^56,57^

**Fig. 8:**
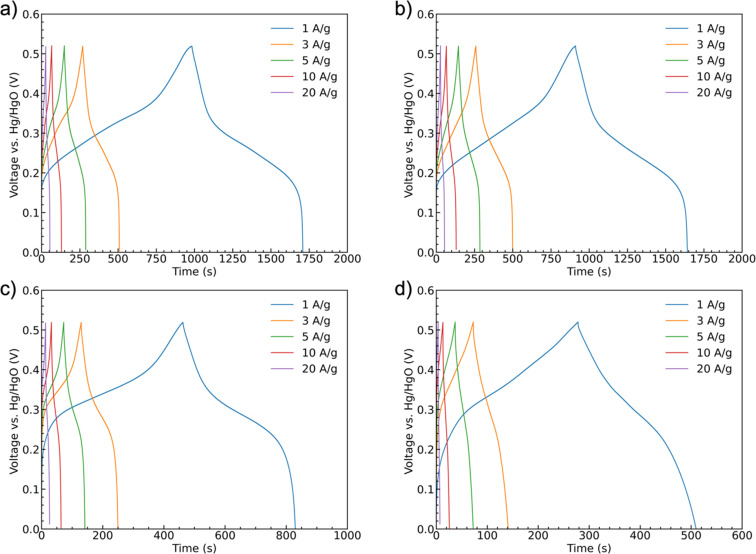
Galvanostatic charge–discharge curves at current densities of 1 A g^−1^, 3 A g^−1^, 5 A g^−1^, 10 A g^−1^, and 20 A g^−1^ of samples: (a) as-synthesized CoMoO_4_, (b) 200 °C CoMoO_4_, (c) 350 °C CoMoO_4_, (d) 450 °C CoMoO_4_.

Galvanostatic charge/discharge curves were recorded for the samples at scan rates of 1 A g^−1^, 3 A g^−1^, 5 A g^−1^, 10 A g^−1^, and 20 A g^−1^ up to 0.52 V, Fig. 8. All of the curves show similar trends with a shallower initial charging gradient at lower potentials. This gradient increases once the accessible sites of the first oxidation state are fully occupied until the charging is stopped. The discharge curves have a shallower shape indicating battery-like behavior. The potential ranges for these shifts in slope are generally consistent with the peak positions observed in the CV scans. Unlike a pseudocapacitive material as MnO_2_, which has a rectangular shape CV curve, CoMoO_4_ does not have constant capacitance over the whole potential window.^58^ Therefore, instead of capacitance, capacity was used to represent the charge storage ability of the CoMoO_4_ samples. Fitting to the standard model, eqn (S3),† the weight-specific capacities of as-synthesized CoMoO_4_, 200 °C CoMoO_4_, 350 °C CoMoO_4_, and 450 °C CoMoO_4_ are 724.9, 731.7, 367.2 and 231.7 C g^−1^ at the current density of 1 A g^−1^, respectively, confirming the capacity trends expected from the CV data. The as-synthesized hydrate-phase CoMoO_4_ exhibited superior specific capacity compared to similar materials (Table S4†).

**Fig. 9:**
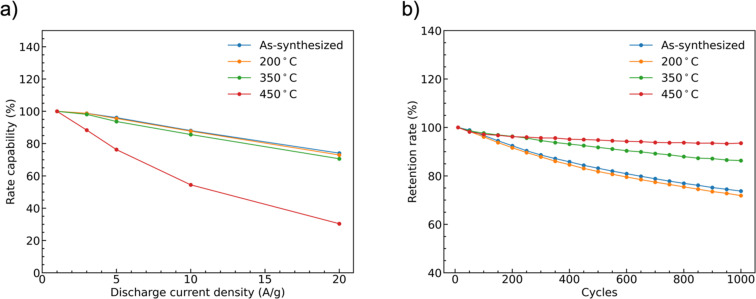
(a) The rate capability of samples as a function of mass-normalized current density normalized to data collected at 1 A g^−1^, and (b) capacity retention at 10 A g^−1^ as a function of the number of charge and discharge cycles.

The rate capability of supercapacitor electrodes at high charge and discharge current density is important for real applications. Fig. 9(a) shows the trend of the specific capacities of the four CoMoO_4_ electrodes at scan rates from 1 A g^−1^ to 20 A g^−1^. The capacities decreased as current density increased because of decreased reactive materials involved in the redox reactions.^32^ The as-synthesized CoMoO_4_, 200 °C CoMoO_4_, and 350 °C CoMoO_4_ exhibited a similar trend of rate capability. The 450 °C CoMoO_4_ had the worst performance among all CoMoO_4_ samples and only had a retention rate of 30% at a high current density of 20 A g^−1^.

The long-term cycle stability of the CoMoO_4_ electrodes was studied at a current density of 10 A g^−1^ for 1000 cycles, Fig. 9b. After 1000 cycles, the specific capacities of the as-synthesized, 200 °C, 350 °C, and 450 °C CoMoO_4_ electrodes were 74%, 72%, 86%, and 94% of the initial capacities, respectively.

**Fig. 10:**
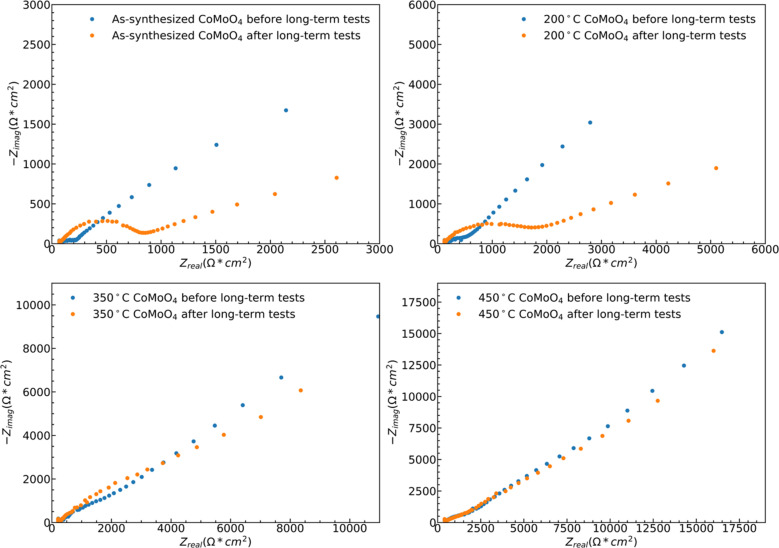
ESCA normalized EIS spectra of all samples before and after 1000-cycle long-term testing.

While both hydrate phase samples show relatively poor cycling stability, no significant morphology changes were observed by SEM, Fig. S5.† Some restructuring is evident upon measuring the ECSA, Fig. S6.† The ECSA was calculated based on eqn S4,† and the ECSA of as-synthesized, 200 °C, 350 °C, and 450 °C CoMoO_4_ before the long-term test are 29.5, 57.5, 105.5 and 186.5 cm^2^, respectively. The ECSA of the as-synthesized material remains relatively unchanged upon 1000 cycles, increasing from 29.5 to 31.25 cm^2^, and that of the 200 °C annealed sample decreases from 57.5 to 41.25 cm^2^. The ECSA-area normalized EIS analysis reveals two dominant processes: a high-frequency arc attributable to a charge transfer process, likely surface kinetics, and a lower-frequency arc attributable to diffusion, Fig. 10. The higher frequency arc of the hydrate phase is smaller than that of the β phase, indicating that the hydrate phase has a smaller charge transfer resistance, which may partially explain why the hydrate phase exhibits higher capacitance. After cycling, both hydrate-phase samples show clear increases in the magnitude of the higher frequency arc, attributed to degradation in surface kinetics.^41^

The 350 °C β-phase material undergoes ECSA loss in a ratio that is close to that of the lost capacity, Fig. S6,† but again shows no clear structural change in SEM, Fig. S5.† This indicates that the degradation is likely due to the loss of surface area without bulk structural change. This loss of active surface is confirmed by the relatively little change in the ECSA-area normalized impedance spectra after cycling, indicating no change in the operating mechanism Fig. 10. As may be expected from the relatively little change in capacity with cycling, the ECSA, EIS data, and SEM observed morphology change little for the 450 °C β-phase material upon cycling.

## 4. Conclusion

The hydrate-phase CoMoO_4_ and the β-phase CoMoO_4_ nanoplate structures can be synthesized by the hydrothermal reaction and subsequent annealing. The facile transition from the hydrate phase to the β-phase upon heating occurs without significant morphological change. Intriguingly, hydrate-phase CoMoO_4_ electrodes exhibit higher specific capacity than their β-phase counterparts, with rapid surface kinetics. Both the rapid surface kinetics and storage capacity exhibited by the hydrate phase degrade significantly upon long-term cycling, although the mass basis capacity is still substantially greater than that for the β-phase samples. This work suggests that it may be more efficient to use the low-temperature synthesized hydrate-phase CoMoO_4_, which was generally regarded as a precursor of CoMoO_4_, directly as a supercapacitor electrode, particularly if the surface kinetics can be maintained or enhanced.

## Conflicts of interest

There are no conflicts to declare.

## Supplementary Material

RA-014-D3RA05878F-s001

## References

[cit1] Yang Z., Zhang J., Kintner-Meyer M. C. W., Lu X., Choi D., Lemmon J. P., Liu J. (2011). Chem. Rev..

[cit2] Nehrir M. H., Wang C., Strunz K., Aki H., Ramakumar R., Bing J., Miao Z., Salameh Z. (2011). IEEE Trans. Sustain. Energy.

[cit3] Simon P., Gogotsi Y. (2008). Nat. Mater..

[cit4] Frackowiak E. (2007). Phys. Chem. Chem. Phys..

[cit5] Chatterjee D. P., Nandi A. K. (2021). J. Mater. Chem. A.

[cit6] Liu H., Liu X., Wang S., Liu H. K., Li L. (2020). Energy Storage Mater..

[cit7] Lukatskaya M. R., Kota S., Lin Z., Zhao M.-Q., Shpigel N., Levi M. D., Halim J., Taberna P.-L., Barsoum M. W., Simon P., Gogotsi Y. (2017). Nat. Energy.

[cit8] Fleischmann S., Mitchell J. B., Wang R., Zhan C., Jiang D. E., Presser V., Augustyn V. (2020). Chem. Rev..

[cit9] Trasatti S., Buzzanca G. (1971). J. Electroanal. Chem..

[cit10] Zheng J. P., Jow T. R. (1995). J. Electrochem. Soc..

[cit11] Jow T. R., Zheng J. P. (1998). J. Electrochem. Soc..

[cit12] Conway B. E. , Electrochemical Supercapacitors: Scientific Fundamentals and Technological Applications, Springer New York, New York, 1999

[cit13] Ghodbane O., Pascal J. L., Favier F. (2009). ACS Appl. Mater. Interfaces.

[cit14] Liu R., Zhou A., Zhang X., Mu J., Che H., Wang Y., Wang T. T., Zhang Z., Kou Z. (2021). Chem. Eng. J..

[cit15] Toupin M., Brousse T., Bélanger D. (2004). Chem. Mater..

[cit16] Lee H. Y., Goodenough J. B. (1999). J. Solid State Chem..

[cit17] Liu K. C., Anderson M. A. (1995). Mater. Res. Soc. Symp. Proc..

[cit18] Yi T. F., Wei T. T., Mei J., Zhang W., Zhu Y., Liu Y. G., Luo S., Liu H., Lu Y., Guo Z. (2020). Adv. Sustainable Syst..

[cit19] Vijayakumar S., Kiruthika Ponnalagi A., Nagamuthu S., Muralidharan G. (2013). Electrochim. Acta.

[cit20] Godillot G., Taberna P. L., Daffos B., Simon P., Delmas C., Guerlou-Demourgues L. (2016). J. Power Sources.

[cit21] Kumar R., Kim H. J., Park S., Srivastava A., Oh I. K. (2014). Carbon.

[cit22] Zeng Y., Yu M., Meng Y., Fang P., Lu X., Tong Y. (2016). Adv. Energy Mater..

[cit23] Augustyn V., Simon P., Dunn B. (2014). Energy Environ. Sci..

[cit24] Liu M. C., Bin Kong L., Lu C., Li X. M., Luo Y. C., Kang L. (2013). Mater. Lett..

[cit25] Li P., Ruan C., Xu J., Xie Y. (2020). Electrochim. Acta.

[cit26] Qian Y., Zhang J., Jin J., Yang S., Li G. (2022). ACS Appl. Energy Mater..

[cit27] Chi K., Zhang Z., Lv Q., Xie C., Xiao J., Xiao F., Wang S. (2017). ACS Appl. Mater. Interfaces.

[cit28] Kazemi S. H., Tabibpour M., Kiani M. A., Kazemi H. (2016). RSC Adv..

[cit29] Xu Z., Li Z., Tan X., Holt C. M. B., Zhang L., Amirkhiz B. S., Mitlin D. (2012). RSC Adv..

[cit30] Yu X., Lu B., Xu Z. (2014). Adv. Mater..

[cit31] Li W., Wang X., Hu Y., Sun L., Gao C., Zhang C., Liu H., Duan M. (2018). Nanoscale Res. Lett..

[cit32] Guo D., Zhang H., Yu X., Zhang M., Zhang P., Li Q., Wang T. (2013). J. Mater. Chem. A.

[cit33] Wang J., Chang J., Wang L., Hao J. (2018). Ionics.

[cit34] Veerasubramani G. K., Krishnamoorthy K., Kim S. J. (2016). J. Power Sources.

[cit35] Eda K., Uno Y., Nagai N., Sotani N., Whittingham M. S. (2005). J. Solid State Chem..

[cit36] Zang M., Xu N., Cao G., Chen Z., Cui J., Gan L., Dai H., Yang X., Wang P. (2018). ACS Catal..

[cit37] Zhang Y., Guo H., Yuan P., Pang K., Cao B., Wu X., Zheng L., Song R. (2019). J. Power Sources.

[cit38] Rodriguez J. A., Chaturvedi S., Hanson J. C., Albornoz A., Brito J. L. (1998). J. Phys. Chem. B.

[cit39] Dam D. T., Huang T., Lee J. M. (2017). Sustainable Energy Fuels.

[cit40] Villa P. L., Trifirò F., Pasquon I. (1974). React. Kinet. Catal. Lett..

[cit41] Nti F., Anang D. A., Han J. I. (2018). J. Alloys Compd..

[cit42] Hu X., Zhang W., Liu X., Mei Y., Huang Y. (2015). Chem. Soc. Rev..

[cit43] Cai D., Liu B., Wang D., Liu Y., Wang L., Li H., Wang Y., Wang C., Li Q., Wang T. (2014). Electrochim. Acta.

[cit44] Bajdich M., García-Mota M., Vojvodic A., Nørskov J. K., Bell A. T. (2013). J. Am. Chem. Soc..

[cit45] Gutierrez A., Benedek N. A., Manthiram A. (2013). Chem. Mater..

[cit46] Wakihara M. and Yamamoto O., Lithium-Ion Batteries: Fundamentals and Performance, John Wiley & Sons, Weinheim, 2008

[cit47] Padhi A. K., Nanjundaswamy K. S., Goodenough J. B. (1997). J. Electrochem. Soc..

[cit48] Shao H., Lin Z., Xu K., Taberna P. L., Simon P. (2019). Energy Storage Mater..

[cit49] Lindström H., Södergren S., Solbrand A., Rensmo H., Hjelm J., Hagfeldt A., Lindquist S. E. (1997). J. Phys. Chem. B.

[cit50] Pan H., Ellis J. F., Li X., Nie Z., Chang H. J., Reed D. (2019). ACS Appl. Mater. Interfaces.

[cit51] Sharma G. P., Vikas, Pala R. G. S., Sivakumar S. (2021). Energy Fuels.

[cit52] Liu T. -C., Pell W. G., Conway B. E., Roberson S. L. (1998). J. Electrochem. Soc..

[cit53] Augustyn V., Simon P., Dunn B. (2014). Energy Environ. Sci..

[cit54] Reddy G. R., Dillip G. R., Manjunath G. L., Joo S. W. (2022). J. Electrochem. Soc..

[cit55] Prasad K., Sreekanth T. V. M., Yoo K., Kim J. (2023). J. Alloys Compd..

[cit56] Reddy G. R., Dillip G. R., Manjunath G. L., Joo S. W. (2022). J. Electrochem. Soc..

[cit57] Malavekar D. B., Kale S. B., Lokhande V. C., Patil U. M., Kim J. H., Lokhande C. D. (2020). J. Phys. Chem. C.

[cit58] Brousse T., Belanger D., Long J. W. (2015). J. Electrochem. Soc..

