# Supplementary material for: The influence of crystal structures on the performance of CoMoO4 battery-type supercapacitor electrodes
Source: RSC Adv. 2024 Mar 11;14(12):8251–9. doi: 10.1039/d3ra05878f (PMC10925852; doi:10.1039/d3ra05878f)
Supplement: RA-014-D3RA05878F-s001 [file RA-014-D3RA05878F-s001.pdf]

Electronic Supplementary Material (ESI) for RSC Advances.
This journal is © The Royal Society of Chemistry 2024

Supplementary Information

The Influence of Crystal Structure on the Performance of $CoMoO_4$ Battery-Type Supercapacitor Electrodes

Kunli Yang[1], Joseph P. Cline[2], Bohyeon Kim[1], Christopher J. Kiely[1,2], Steven McIntosh[1*]

[1] Department of Chemical and Biomolecular Engineering, Lehigh University, Bethlehem, PA 18015, USA

[2] Department of Materials Science and Engineering. Lehigh University, Bethlehem, PA 18105, USA

*mcintosh@lehigh.edu

1. Supplementary Figures

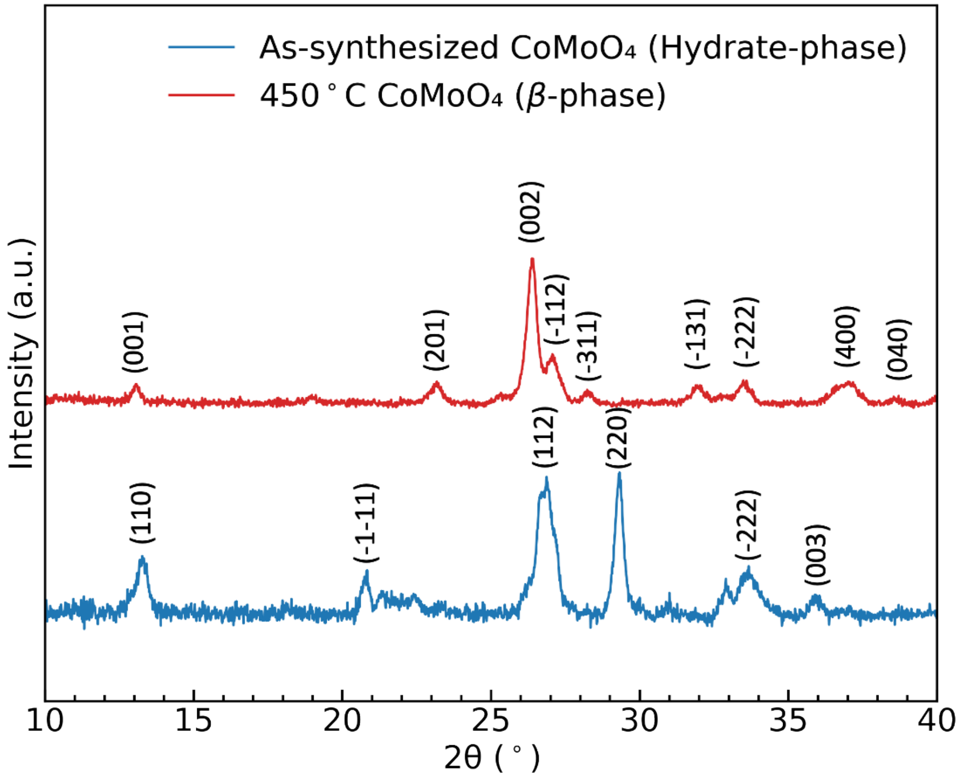

Figure S1: X-ray diffraction patterns of the as-synthesized and 450˚C $CoMoO_4$.

The interlayer spacing is calculated by Bragg's law (Equation S1) based on the data extracted from the XRD. The interlayer spacing of the (-1-11) (112) and (220) planes of the hydrate-phase $CoMoO_4$ is 0.426nm, 0.315nm and 0.301nm, respectively. The interlayer spacing of the (001) and (002) plane of the β-phase $CoMoO_4$ is 0.590 nm and 0.331nm, respectively. The interlayer spacing matches with the reference, and confirms the more open structure of the hydrate phase [1-3].

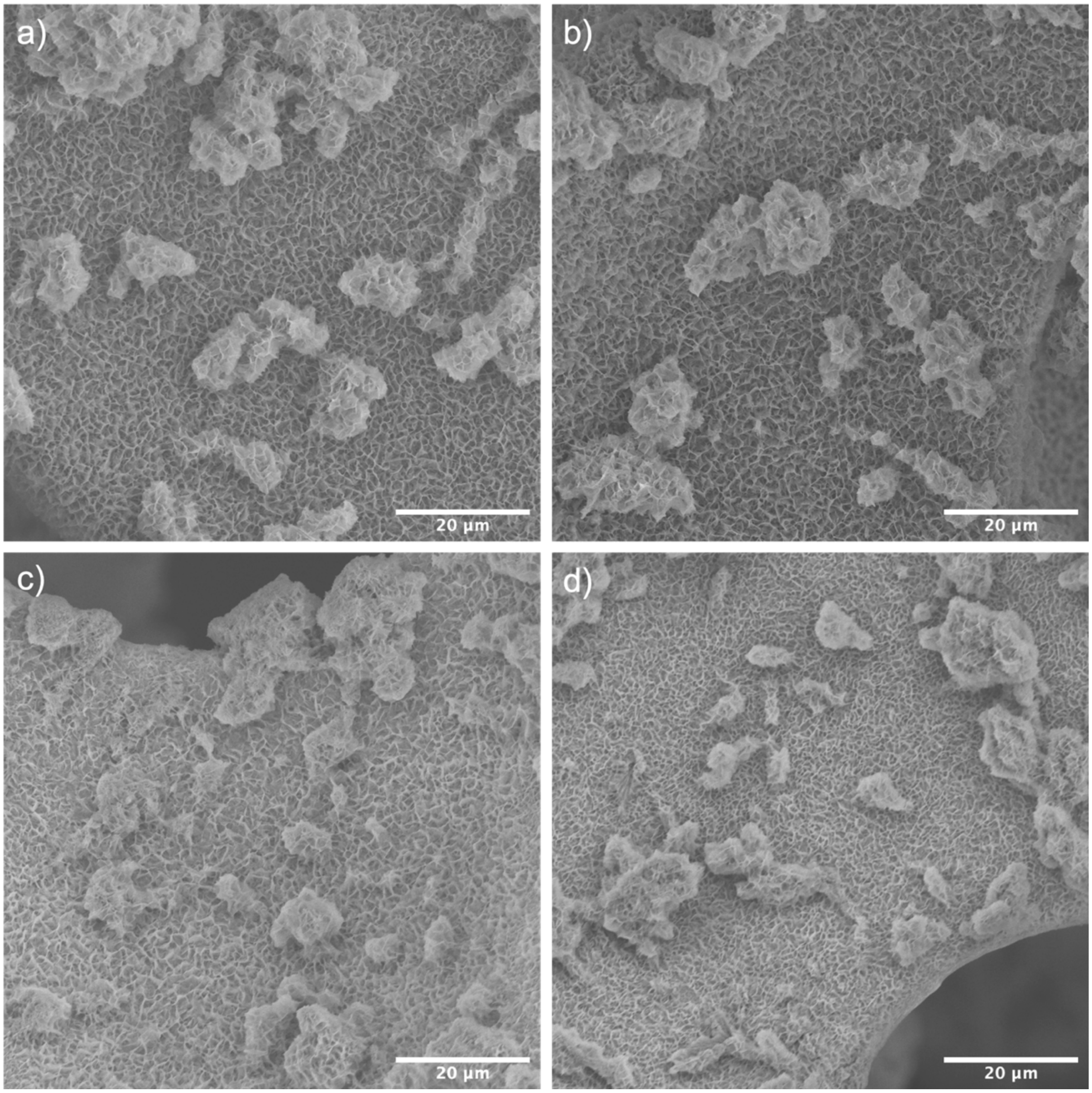

Figure S2: Low-magnification SEM images of the a) as-synthesized, b) 200°C, c) 350°C, and d) 450°C samples.

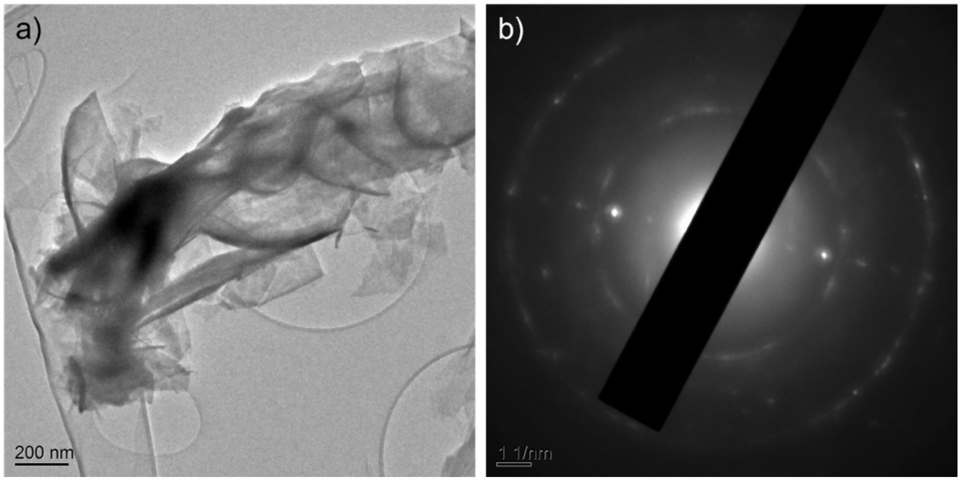

Figure S3: a) TEM image of the as-synthesized material showing the nanoplate morphology, and b) the corresponding electron diffraction pattern fitting to the hydrate structure.

A small amount of α-phase $CoMoO_4$ beyond the detection limit of XRD was detected.

Table S1: Measured plane spacings from electron diffraction patterns in the hydrate phase for $CoMoO_4$. Note that errors were calculated using the third significant digits in spacings but only two are reported herein to reflect accuracy.

Hydrate phase	Experimental (nm)	Hydrate Phase	% Error
(0 0 1)	0.91	0.91	0.44%
(1 1 1)	0.48	0.51	-5.87%
(1 1 3)	0.28	0.29	-2.75%

Table S2: Measured plane spacings from electron diffraction patterns in the α phase for $CoMoO_4$. Note that errors were calculated using the third significant digits in spacings but only two are reported herein to reflect accuracy.

α phase	Experimental (nm)	Alpha Phase	% Error
(0 0 1)	0.73	0.72	1.5%
(0 2 0)	0.46	0.45	2.9%
($\bar{2}$ 0 2)	0.36	0.36	1.7%

Table S3. Fitted b values of redox reaction peaks of all the samples:

	Oxidation 1	Oxidation 2
As-synthesized	0.82	0.82
200°C	0.81	0.92
350°C		0.89
450°C	0.74	0.99

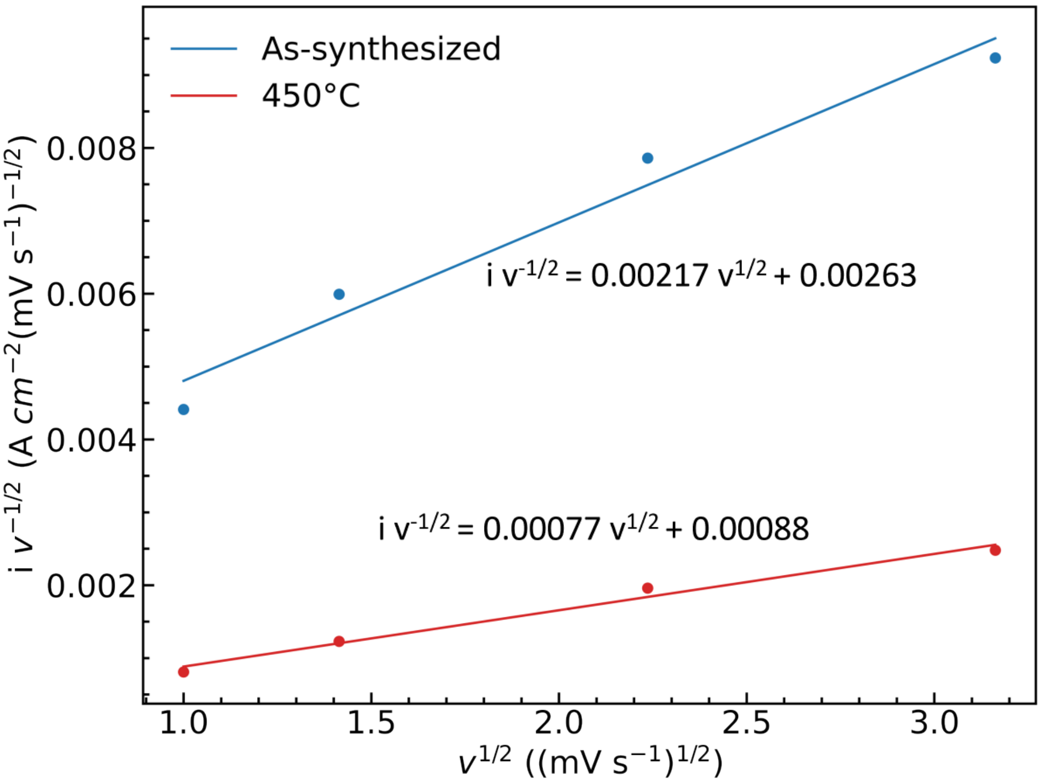

Figure S4: Plots of i $v^{-1/2}$ and $v^{1/2}$ of as-synthesized and 450°C $CoMoO_4$.

The values of k_1 and k_2 can be obtained by plotting and fitting $i/v^{1/2}$ vs. $v^{1/2}$.

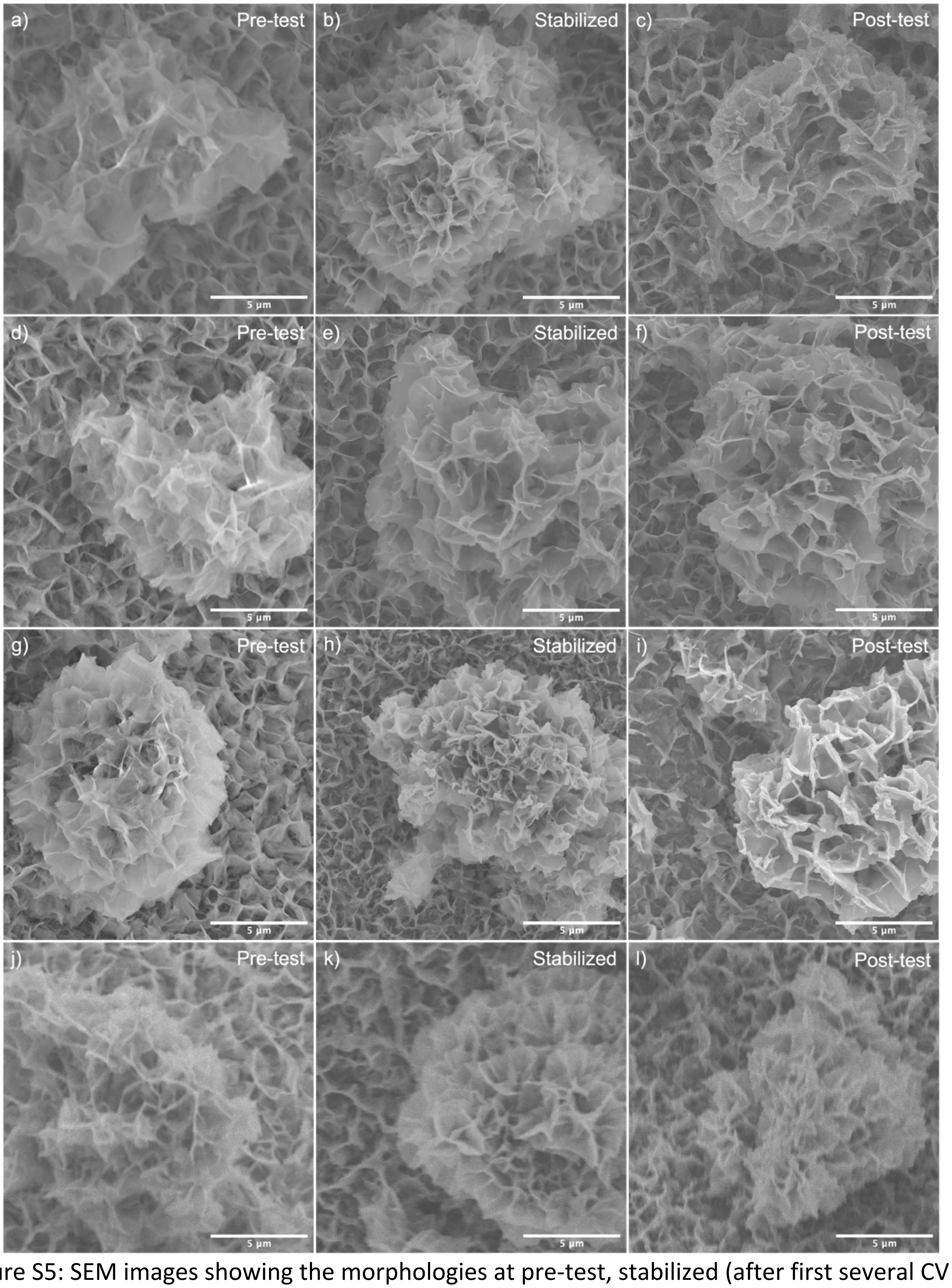

Figure S5: SEM images showing the morphologies at pre-test, stabilized (after first several CV cycles until the CV curve is stable), and post-test (after 1000-cycle GCD long-term test) stages in as-synthesized (a, b, c), 200°C (d, e, f), 350°C (g, h, i), and 450°C $CoMoO_4$ (j, k, l)

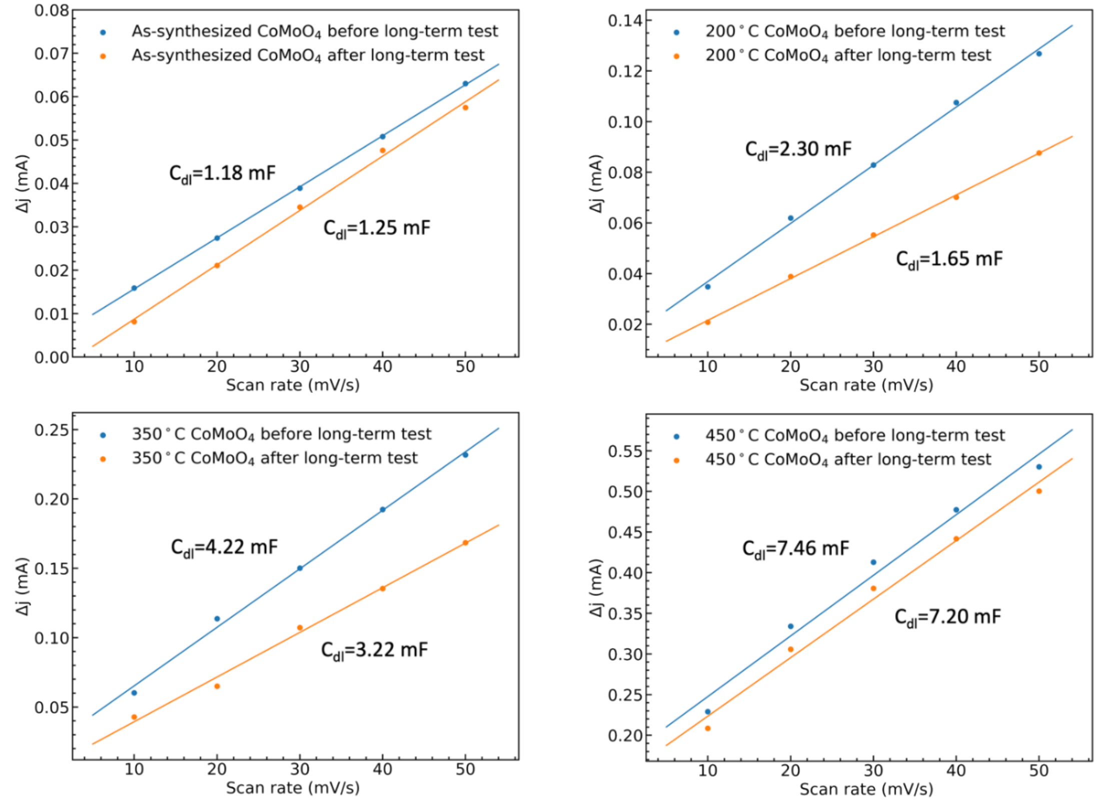

Figure S6: Data used to calculate the ECSA (electrochemical surface area) of as-synthesized, 200°C, 350°C, and 450°C $CoMoO_4$ before and after 1000 cycle long-term tests.

The ECSA was calculated based on Equation S4, and the ECSA of as-synthesized, 200°C, 350°C, and 450°C $CoMoO_4$ before the long-term test are 29.5, 57.5, 105.5 and 186.5 cm^2, respectively.

Table S4: Comparison between the hydrate-phase $CoMoO_4$ and similar materials.

Electrode	Current density	Capacity	Refs.
Hydrate-phase $CoMoO_4$	1 A g^{-1}	724.9 C g^{-1}	This work
$CoMoO_4$/rGO/PANI	1 A g^{-1}	630 C g^{-1}	[4]
$CoMoO_4$@RGO	1 A g^{-1}	428 C g^{-1}	[5]
1D $CoMoO_4$ nanorods	5 mA cm^{-2}	658 C g^{-1}	[6]
2D $CoMoO_4$ nanosheets	1 mA cm^{-2}	77 C g^{-1}	[7]
3D $CoMoO_4$/rGO	1 A g^{-1}	400 C g^{-1}	[8]
$CoMoO_4$/Co_3O_4	1 A g^{-1}	425 C g^{-1}	[9]
MnO_2/NiCo-LDH	1 A g^{-1}	173.6 C g^{-1}	[10]
NiCo-LDH/NCF	1 A g^{-1}	750 C g^{-1}	[11]
$NiCo_2O_4$@MnO_2/CNT	5 A g^{-1}	524 C g^{-1}	[12]
Ni–Co–P/C	1 A g^{-1}	775.7 C g^{-1}	[13]

The result suggests that the hydrate-phase $CoMoO_4$ without annealing exhibited superior capacity among the similar materials. The specific capacity of the hydrate-phase is the highest among other published $CoMoO_4$ electrodes at the same current density.

2. Supplementary Calculation Details

Interlayer Spacing

The interlayer spacing of different planes of the hydrate phase and β phase is calculated by Bragg's law:

$$d = \frac{\lambda}{2\sin\theta}$$ Equation S1

d (nm) is the spacing between planes in the crystal lattice, λ is the wavelength of the X-ray and θ (°) is the angle of incidence.

Cyclic Voltammetry Fitting

The peak current (i) and the scan rate (mV/s) can be fitted by the following equation [14, 15]:

$$i = a\vartheta^{b}$$ Equation S2

a and b are adjustable parameters between 0.5 and 1.

The value of a and b depends on the charge storage mechanisms and reaction kinetics [16]. If the value of b is close to 0.5, the process is assigned to a a diffusion-controlled process, and if the value of b is close to 1, the process is assigned to a a diffusion-free process.

Capacity

From the GCD curves, the mass-specific capacity of the electrode can be calculated by the following equation:

$$Q = I \times t$$ Equation S3

Q is the capacity (C/g), I (A/g) is the discharging current density, t (s) is the discharging time.

Electrochemical Surface Area (ECSA)

Quantifying the ECSA of an electrode helped in knowing the area of the reacting interface, therefore, normalizing current by ECSA was an important method to assess the intrinsic electrochemical activity of an electrode. The ECSA was calculated by the following equation:

$$ECSA = \frac{C_{DL}}{C_s}$$ Equation S4

The C_{DL} is the double-layer capacitance of the electrode. C_S is the specific capacitance, and in our case, because the experiments were performed in an alkaline electrolyte, Cs was chosen as

0.04 mF/cm^2 [17]. C_{DL} of an electrode was estimated by CV scan in a small potential window in a non-Faradaic region [18]. To gain accurate and comparable estimation of the C_{DL} of the four samples, CV scan was first performed at 10mV/s from 0V to 0.6 V (vs. Hg/HgO) to make sure the samples were in the same condition before the ECSA estimation. Then, the CV scanning was performed at the same potential range for all $CoMoO_4$ samples.

Reference

(1) M. Zang, N. Xu, G. Cao, Z. Chen, J. Cui, L. Gan, H. Dai, X. Yang and P. Wang, *ACS Catal.*, 2018, **8**, 5062–5069.

(2) Y. Zhang, H. Guo, P. Yuan, K. Pang, B. Cao, X. Wu, L. Zheng and R. Song, *J. Power Sources*, 2019, **442**, 227252.

(3) K. Eda, Y. Uno, N. Nagai, N. Sotani and M. S. Whittingham, *J. Solid State Chem.*, 2005, **178**, 2791–2797.

(4) H. M. Fahad, R. Ahmad, F. Shaheen, S. M. Ali and Q. Huang, *Electrochim. Acta*, 2023, **471**, 143393.

(5) L. Jinlong, Y. Meng, K. Suzuki and H. Miura, *Microporous Mesoporous Mater.*, 2017, **242**, 264–270.

(6) G. Cheng, C. Si, J. Zhang, Y. Wang, W. Yang, C. Dong and Z. Zhang, *J. Power Sources*, 2016, **312**, 184–191.

(7) Y. Zhao, Z. Liu, W. Gu, Y. Zhai, Y. Teng and F. Teng, *Nanotechnology*, 2016, **27**, 505401.

(8) C. Wang, Z. Guan, M. Wei, S. Yu, R. Sun and C. P. Wong, *Proc. - 2018 19th Int. Conf. Electron. Packag. Technol. ICEPT 2018*, 2018, 1297–1300.

(9) M. Zhou, F. Lu, X. Shen, W. Xia, H. He and X. Zeng, *J. Mater. Chem. A*, 2015, **3**, 21201–21210.

(10) L. Liu, L. Fang, F. Wu, J. Hu, S. Zhang, H. Luo, B. Hu and M. Zhou, *J. Alloys Compd.*, 2020, **824**, 153929.

(11) Y. Liu, Y. Wang, C. Shi, Y. Chen, D. Li, Z. He, C. Wang, L. Guo and J. Ma, *Carbon N. Y.*, 2020, **165**, 129–138.

(12) S. Karmakar, R. Boddhula, B. Sahoo, B. Raviteja and D. Behera, *J. Solid State Chem.*, 2019, **280**, 121013.

(13) Q. Zhou, Y. Gong and K. Tao, *Electrochim. Acta*, 2019, **320**, 134582.

(14) V. Augustyn, J. Come, M. A. Lowe, J. W. Kim, P.-L. Taberna, S. H. Tolbert, H. D. Abruña, P. Simon and B. Dunn, *Nat. Mater.*, 2013, **12**, 518–522.

(15) T. -C. Liu, W. G. Pell, B. E. Conway and S. L. Roberson, *J. Electrochem. Soc.*, 1998, **145**, 1882.

(16) H. Pan, J. F. Ellis, X. Li, Z. Nie, H. J. Chang and D. Reed, *ACS Appl. Mater. Interfaces*, 2019, **11**, 37524–37530.

(17) J. Wang, H. Xuan, L. Meng, X. Liang, Y. Li, J. Yang and P. Han, Int. J. Hydrog. Energy, 2023, **48**, 8144-8155.

(18) B. Fei, Z. Chen, Y. Ha, R. Wang, H. Yang, H. Xu and R. Wu, J. Chem. Eng., 2020, **394**, 124926.
